# Risk Factors for Asthma-Related Healthcare Use: Longitudinal Analysis Using the NHI Claims Database in a Korean Asthma Cohort

**DOI:** 10.1371/journal.pone.0112844

**Published:** 2014-11-14

**Authors:** Taehoon Lee, Jinhee Kim, Sujeong Kim, Kyoungjoo Kim, Yunjin Park, Yuri Kim, Yoon Su Lee, Hyouk-Soo Kwon, Sae-Hoon Kim, Yoon-Seok Chang, You Sook Cho, An-Soo Jang, Jung-Won Park, Dong-Ho Nahm, Ho-Joo Yoon, Sang-Heon Cho, Young-Joo Cho, Byoung Whui Choi, Hee-Bom Moon, Tae-Bum Kim, for the COREA study group

**Affiliations:** 1 Department of Allergy and Clinical Immunology, Asan Medical Center, University of Ulsan College of Medicine, Seoul, Korea; 2 Department of Internal Medicine, Ulsan University Hospital, University of Ulsan College of Medicine, Ulsan, Korea; 3 National Evidence-based Healthcare Collaborating Agency, Seoul, Korea; 4 Department of Nursing, College of Medicine, Chosun University, Gwangju, Korea; 5 Department of Internal Medicine, Kyungpook National University School of Medicine, Daegu, Korea; 6 Department of Statistics, Dongguk University, Seoul, Korea; 7 Department of Internal Medicine, Seoul National University College of Medicine, Seoul, Korea; 8 Department of Internal Medicine, Soonchunhyang University Bucheon Hospital, Bucheon, Korea; 9 Department of Internal Medicine, College of Medicine, Yonsei University, Seoul, Korea; 10 Department of Internal Medicine, College of Medicine, Ajou University, Suwon, Korea; 11 Department of Internal Medicine, College of Medicine, Hanyang University, Seoul, Korea; 12 Department of Internal Medicine, College of Medicine, Ewha Womans University, Seoul, Korea; 13 Department of Internal Medicine, College of Medicine, Chung-Ang University, Seoul, Korea; Cincinnati Children's Hospital Medical center, United States of America

## Abstract

**Background:**

Though insurance claims data are useful for researching asthma, they have important limitations, such as a diagnostic inaccuracy and a lack of clinical information. To overcome these drawbacks, we used the novel method by merging the clinical data from our asthma cohort with the National Health Insurance (NHI) claims data.

**Methods and Results:**

Longitudinal analysis of asthma-related healthcare use from the NHI claims database, merged with data of 736 patients registered in a Korean asthma cohort, was conducted for three consecutive years from registration of the cohort. Asthma-related asthma healthcare referred to outpatient and emergency department visits, hospitalizations, and the use of systemic corticosteroids. Univariate and multivariate logistic regression analysis was used to evaluate risk factors for asthma-related healthcare. Over three years after enrollment, many patients changed from tertiary to primary/secondary hospitals with a lack of maintenance of inhaled corticosteroid-based controllers. An independent risk factor for emergency visits was a previous history of asthma exacerbation. In hospitalizations, old age and Asthma Control Test (ACT) score variability were independent risk factors. An independent risk factor for per person cumulative duration of systemic corticosteroids was the FEV_1_ (Forced expiratory volume in one second)%. The use of systemic corticosteroids was independently associated with being female, the FEV_1_%, and ACT score variability.

**Conclusion:**

We found that old age, being female, long-standing asthma, a low FEV_1_%, asthma brittleness, asthma drug compliance, and a history of asthma exacerbation were independent risk factors for increased asthma-related healthcare use in Korea.

## Introduction

In order to establish an asthma control strategy, it is important to evaluate patterns of healthcare use that can reveal risk factors for poor control and frequent asthma exacerbation. However, many researchers have failed to obtain comprehensive data of healthcare use from asthma patients due to considerable loss of patients to follow-up to their studies, potentially resulting in a selection bias; it is crucial to investigate healthcare use data without patient loss in order to unravel the risk factors for poor control and asthma exacerbation.

For this reason, numerous studies have used health insurance claims data to examine asthma prevalences, [1]–[3] socioeconomic costs, [2], [4] healthcare use, [5], [6] controller prescriptions, [7], [8] comorbidities, [9] and the relationships between certain medications and asthma outcomes. [6], [10]–[12] Korea, for example, has a single National Health Insurance (NHI) system that covers almost the entire population, and whose claims records permit the analysis of the prevalence of adult asthma, the patterns of asthma-related healthcare use, and medication prescriptions.

Nonetheless, insurance claims data have important limitations. For instance, the diagnoses in claims data are not always accurate, because many physicians may diagnose asthma to claim medical costs, despite the lack of a positive result in a confirmatory test for suspected asthma. In addition, claims data contain little clinical information on each patient. Therefore, we could not address the exact clinical risk factors for asthma-related healthcare utilization with sole insurance claims data [5].

Thus, we merged the clinical data from our asthma cohort [13] with that of the asthma-related healthcare use from the NHI claims in order to analyze the healthcare use of asthmatics in the real world. This novel data-merging method between clinical and NHI claims dataset, to the best of our knowledge, has not been used in the past in any research field including asthma. These complementary data enabled us to approach more closely to the real-world situation in patients with definite asthma.

The aim of this study was to determine the risk factors for asthma-related healthcare use by longitudinal analysis of the NHI claims data connected with the asthma cohort in our country. In addition, in order to evaluate asthma management related to the type of hospital, drug-prescription pattern and healthcare utilization were investigated according to type of hospital.

## Methods

### Study population

In this study, the data of 736 patients from the COREA (COhort for Reality and Evolution of adult Asthma in Korea) cohort [13], [14] (See Methods and Results S1) registered between January 2006 and December 2007 were linked to the NHI claims database. Each individual’s clinical information from the COREA cohort and the asthma-related healthcare use from the claims data were thoroughly investigated for a duration of 3 consecutive years from the time of the COREA enrollment, which was considered the index period. For example, if a patient’s enrollment date was 9-June-2007, the patient’s NHI claims data were extracted from 9-June-2007 to 8-June-2010.

We presented baseline characteristics of the subjects included in the COREA cohort (4 clusters) and NHI claims database as Table S1 and S2 in File S1 from our previous studies [15], [16].

### Ethics

This study was approved by the Ethics Committee of the National Evidence-Based Healthcare Collaborating Agency (NECA) (PIRB11-024-1). All study participants were fully informed of the study protocol and provided written, signed statements of informed consent.

### Data sources and study design

NHI claims data include site of care as well as diagnoses (ICD-10 codes), prescribed drugs and procedures for medical services. Data for asthma-related healthcare were specifically extracted from the NHI claims of the study population. Asthma-related healthcare use encompassed claims that were marked with ICD-10 codes J45.x–J46.x for the principal or additional diagnoses of asthma, and in which at least one of the following asthma medications were prescribed: inhaled corticosteroids (ICSs), long-acting β2-agonists (LABAs), ICS and LABA combined in a single inhaler (ICSs/LABAs), oral leukotriene antagonists, short-acting β2-agonists (SABAs), long-acting muscarinic antagonists (LAMAs), short-acting muscarinic antagonists (SAMAs), systemic β-agonists, systemic corticosteroids, and theophylline derivatives. The definition also included claims that were checked by at least one of the following tests for the diagnosis of asthma: spirometry (with or without bronchodilator response, BDR) and the bronchial provocation test.

### Measures

#### Outcome variables: Asthma-related healthcare use

Asthma-related healthcare use was measured by the numbers of outpatient visits, hospitalizations, and emergency department visits, and the presence and number of prescribed days of systemic corticosteroids. Outpatient visits were defined as claims produced by outpatient clinic with asthma related ICD-10 codes and either asthma medications or asthma related tests (see the above “Data sources and study design”), thus it included scheduled visits as well as unscheduled visits such as asthma exacerbations. Hospitalizations and emergency department visits were defined when claims were produced with patient’s admission and emergency department visits (respectively) with asthma related ICD-10 codes and either asthma medications or asthma related tests. For hospitalizations and emergency department visits, only claims that involved the prescription of oral or injected systemic corticosteroids were defined as asthma-related claims in order to exclude those claims in which asthma was not the principal reason for the visit. The presence and number of prescribed days of systemic corticosteroids were claims with systemic corticosteroid-prescription with asthma related ICD-10 codes and either asthma medications or asthma related tests.

In addition, the types of medical institutions where patients visited were analyzed. Primary hospitals are outpatient care centers or hospitals with less than 30 beds in general wards, whereas secondary ones have 30–99 inpatient beds. Tertiary ones have 100 or more inpatient beds and more than seven medical departments [17].

#### Clinical phenotypes

Clinical phenotypes included airway reversibility, Asthma Control Test (ACT) score variability, treatability, adherence of inhalers and all medications, and clinical clusters.

Airway reversibility referred to the status of improvement of airway obstruction after asthma treatment, divided into two categories: fixed airway obstruction (FAO) and reversible airway obstruction (RAO). [14] FAO was defined as an FEV_1_ (Forced expiratory volume in one second)/functional volume capacity (FVC) ratio <70% on all pulmonary function tests for the first 12 months despite the use of high-dose ICS/LABA. RAO was defined when an FEV_1_/FVC ratio >70% was shown on at least one pulmonary function test during the index period.

ACT score variability was calculated when a patient’s ACT score was measured more than 3 times during the index period by summing the differences between successive ACT scores during a 3-year period and dividing by whole ACT score measurement number minus one. Thus, a higher value of the ACT score variability indicates a more ‘brittle’ (labile) asthma.

Patients who did not reach an acceptable level of control, even by treatment of step 4 of Global Initiative of Asthma (GINA) guidelines, [18] were considered to have difficult-to-treat asthma. Adherence to medication was measured by patients with a 100 mm-visual analogue scale (VAS): 100 was the highest compliance, and 0 was the lowest. High adherence to asthma medication was defined as a VAS score >75/100. For each of the drugs and inhalants, the average 3-year compliance was calculated. Cluster analysis of the COREA cohort indicated four asthma subtypes: (i) smoking asthma, (ii) severe obstructive asthma, (iii) early-onset atopic asthma, and (iv) late-onset mild asthma [16].

#### Baseline clinical characteristics, Prescriptions, Adherence to the cohort and Statistical analysis

See Methods and Results S1.

## Results

### Baseline clinical characteristics

The mean participant age was 49.9 years, and there was a slight female predominance (55%) (Table 1). The ratios of atopy, rhinosinusitis, and family history of allergic diseases were 0.52, 0.67 and 0.47, respectively. Thirty-nine percent had experienced an exacerbation of asthma. Eighty-five percent had been exposed to smoking. The percentages of the FEV_1_% predicted and the FEV_1_/FVC ratio were 81.5% and 72.6% of the average, respectively. Severe asthma accounted for 29% of the total according to GINA guidelines [19].

**Table 1:** Baseline clinical characteristics of the COREA cohort.

Total number of patients (n = 736)	
Age (years; mean ± SD)	49.9±15.4
Sex	
Male	45%
Female	55%
BMI (kg/m^2^; mean ± SD)	24.2±3.3
Atopy	
Presence	52%
Absence	48%
Rhinosinusitis	
Presence	67%
Absence	33%
Family history of allergic diseases	
Presence	47%
Absence	53%
Duration of asthma (years; mean ± SD)	9.8±11.3
History of exacerbation	
Presence	39%
Absence	61%
Smoking	
Current	15%
Ex-	45%
Non-	40%
Pack-years of smoking in smokers (mean ± SD)	22.4±20.3
Blood eosinophils (% of total WBC; mean ± SD)	7.3±57.5
Sputum eosinophils (% of total WBC; mean ± SD	12.5±17.0
Log IgE (mean ± SD)	2.3±0.7
Initial FEV_1_/FVC (%; mean ± SD)	74.0±12.3
Initial FEV_1_ (predicted %; mean ± SD)	81.5±21.8
BDR (L; mean ± SD of 457 patients)	0.2±0.6
Initial severity of asthma	
Mild intermittent	5%
Mild persistent	19%
Moderate persistent	47%
Severe persistent	29%

Abbreviations: WBC, white blood cell; BMI, Body mass index.

### Asthma-related healthcare use according to baseline clinical characteristics

In patients over 60 years of age, the number of per person outpatient visits was significantly higher and hospitalizations were more common (both p<0.001) than in patients under 60 years of age (Table 2, Table S4 in File S1). Furthermore, the per person cumulative duration of systemic corticosteroids was significantly longer (p = 0.001) and the use of systemic corticosteroids was more frequent (p = 0.016). The number of per person outpatient visits and the use of systemic corticosteroids were more common (p = 0.005 and p = 0.003, respectively) in females. The atopic status was negatively associated with per person outpatient visits (p = 0.022). Absence of rhinosinusitis was associated with poor asthma control with more frequent per person outpatient visits (p = 0.004), more hospitalizations (per person, p = 0.036) and more per person cumulative duration of systemic corticosteroids (p = 0.034). Long-standing asthma (>10 years) was associated with more per person outpatient visits (p = 0.006), hospitalizations (p = 0.007), per person cumulative duration of systemic corticosteroids (p<0.001), and systemic corticosteroid use (p = 0.013). Although non-smokers had more per person outpatient visits (p = 0.026) than those who were exposed to smoking, heavy smoking (≥10 pack-years) was positively associated with the per person cumulative duration of systemic corticosteroids (p = 0.002). A previous history of exacerbation was also associated with poor asthma control with more frequent per person outpatient visits, emergency visits, hospitalizations, and systemic steroid use (all p<0.001). BDR results were positively associated with the per person cumulative duration of systemic corticosteroids (p = 0.008). A low initial FEV_1_/FVC ratio and low FEV_1_% were also significantly associated with poor asthma control with more outpatient visits, emergency department visits, hospitalizations, and systemic corticosteroid use (all p<0.05).

**Table 2:** Asthma-related healthcare use: Positively related, statistically significant (p<0.05) variables.

Per capita frequency of outpatient visits	
Baseline clinical characteristics	Age ≥60 years
	Female
	Absence of atopy
	Absence of rhinosinusitis
	Duration of asthma ≥10 years
	Non-smoker
	Presence of exacerbation
	Initial FEV1/FVC (%) ≤70
	Initial FEV1 (predicted %) ≤60
	Initial severity of asthma: Severe persistent
Clinical phenotypes	Airway reversibility: FAO
	Difficult-to-treat asthma
	Variability of ACT score ≥2
	Compliance of all drugs >75
	Compliance of inhalers >75
	Cluster 2: Severe obstructive asthma
Adherence to the cohort	Cohort-maintenance group
Per capita frequency of emergency department	
Baseline clinical characteristics	Absence of family history of allergic diseases
Per capita frequency of emergency department	
Baseline clinical characteristics	Presence of exacerbation
	Initial FEV1 (predicted %) ≤60
	Initial severity of asthma: Severe persistent
Per capita frequency of hospitalizations	
Baseline clinical characteristics	Absence of rhinosinusitis
	Ex-smoker
Per capita cumulative duration (days) ofhospitalizations	
Baseline clinical characteristics	Initial FEV1/FVC (%) ≤70
Presence of hospitalizations	
Baseline clinical characteristics	Age ≥60 years
	Duration of asthma ≥10 years
	Presence of exacerbation
	Initial FEV1 (predicted %) ≤60
	Initial severity of asthma: Severe persistent
Clinical phenotypes	Difficult-to-treat asthma
	Variability of ACT score ≥2
	Compliance of inhalers >75
Per capita cumulative duration (days) ofsystemic steroids use	
Baseline clinical characteristics	Age ≥60 years
	Absence of rhinosinusitis
	Duration of asthma ≥10 years
	Pack-years of smoking≥10
	Blood eosinophils <5% of total WBC
	BDR: Positive
	Initial FEV1/FVC (%) ≤70
	Initial FEV1 (predicted %) ≤60
	Initial severity of asthma: Severe persistent
Clinical phenotypes	Airway reversibility: FAO
	Difficult-to-treat asthma
	Variability of ACT score ≥2
	Compliance of all drugs >75
	Compliance of inhalers >75
	Cluster 2: Severe obstructive asthma
Presence of systemic steroids use	
Baseline clinical characteristics	Age ≥60 years
	Female
	Duration of asthma ≥10 years
	Non-smoker
	Presence of exacerbation
	Initial FEV1 (predicted %) ≤60
Clinical phenotypes	Difficult-to-treat asthma
Adherence to the cohort	Cohort-maintenance group

### Asthma-related healthcare use according to clinical phenotypes

In patients with FAO, the number of per person outpatient visits was significantly higher (p = 0.002) than in patients with RAO (Table 2, Table S5 in File S1). Furthermore, the per person cumulative duration of systemic corticosteroids was significantly longer (p = 0.002) in patients with FAO. Difficult-to-treat asthma was associated with more per person outpatient visits (p<0.001), hospitalizations (p = 0.011), per person cumulative duration of systemic corticosteroids (p = 0.001), and use of systemic corticosteroids (p = 0.001). A high ACT score variability (≥2) was also associated with poor asthma control with more frequent per person outpatient visits (p<0.001), hospitalizations (p = 0.022), and per person cumulative duration of systemic corticosteroids (p = 0.012). Good drug compliance (≥75) was significantly correlated with increased per person outpatient visits, hospitalizations, and per person cumulative duration of systemic corticosteroids. Among clusters of the COREA cohort, the severe obstructive asthma subtype was associated with the poorest asthma control with the most per person outpatient visits (p = 0.010) and an increased per person cumulative duration of systemic corticosteroids (p<0.001).

### Asthma-related healthcare use according to adherence to the cohort

In patients maintained in the cohort, the number of per person outpatient visits was significantly higher (p<0.001) and the use of systemic corticosteroids was more frequent (p<0.001) (Table 2, Table S6 in File S1). When patients with poor lung function (FAO) dropped out of the cohort, they showed a non-significant trend towards more emergency visits and greater systemic corticosteroid use.

### Multivariate analysis of risk factors for asthma-related healthcare use

Per person outpatient visits were independently associated with the duration of asthma (β = 0.13; 95% CI, 0.01 to 0.25), FEV_1_% (β = −0.06; 95% CI, −0.11 to −0.01), ACT score variability (β = 1.50; 95% CI, 0.83 to 2.16), and inhaler compliance (β = 0.09; 95% CI, 0.04 to 0.14) (Table 3). An independent risk factor for emergency visits was a previous history of asthma exacerbation (OR = 3.35; 95% CI, 1.12 to 10.02) (Table 4). With regard to hospitalizations, age (OR = 1.03; 95% CI, 1.01 to 1.06) and ACT score variability (OR = 1.31; 95% CI, 1.11 to 1.55) were independent risk factors (Table 5). An independent risk factor for an increased per person cumulative duration of systemic corticosteroids was the FEV_1_% (β = −3.05; 95% CI, −4.23 to −1.87) (Table 6). The use of systemic corticosteroids was independently associated with a female gender (OR = 1.76; 95% CI, 1.11 to 2.78), FEV_1_ (OR = 0.98; 95% CI, 0.97 to 0.99), and ACT score variability (OR = 1.41; 95% CI, 1.20 to 1.65) (Table 7).

**Table 3:** Multivariate analysis of risk factors for asthma-related healthcare use: Per person frequency of outpatient visits.

Variable	*Unadjusted*				*Adjusted* 1			
	β	95 % CI		p	β	95% CI		p
Age (per year)	0.15	0.10	0.21	<0.001	0.07	−0.01	0.16	0.076
Sex (female)	2.33	0.70	3.97	0.005	−0.03	−2.51	2.45	0.981
Duration of asthma (per year)	0.12	0.05	0.19	0.001	0.13	0.01	0.25	0.037
BMI (per kg/m^2^)	−0.01	−0.26	0.23	0.918				
History of exacerbation (presence)	3.53	1.86	5.20	<0.001	1.19	−1.28	3.66	0.342
Rhinosinusitis (presence)	−2.59	−4.36	−0.82	0.004	−1.50	−4.00	1.00	0.238
Family history of allergy disease (presence)	−0.40	−2.04	1.24	0.630				
Smoking (≥10 pack-years)	0.17	−1.83	2.17	0.865				
FEV_1_ (per predicted %)	−0.11	−0.15	−0.07	<0.001	−0.06	−0.11	−0.01	0.019
BDR (presence)	2.04	−0.73	4.80	0.148				
Blood eosinophilia (per % of total WBC)	0.00	−0.01	0.02	0.879				
Atopy (presence)	−2.29	−4.41	−0.17	0.035	−0.53	−3.51	2.46	0.729
Follow-up (maintained in the cohort)	3.38	1.68	5.08	0.001	2.58	−0.83	5.98	0.137
Log IgE	−1.83	−3.55	−0.12	0.037	−1.32	−3.34	0.71	0.201
ACT score variability	1.55	1.08	2.01	<0.001	1.50	0.83	2.16	<0.001
Inhaler compliance score	0.08	0.04	0.12	<0.001	0.09	0.04	0.14	<0.001

1 Adjusted for age, sex, and variables that had a p-value <0.1 in the unadjusted model.

Abbreviations: WBC, white blood cell; BMI, Body mass index.

**Table 4:** Multivariate analysis of risk factors for asthma-related healthcare use: Presence of emergency department visits.

Variable	Unadjusted				Adjusted^1^			
	OR	95% CI		p	OR	95% CI		p
Age (per year)	1.01	0.99	1.02	0.258	1.02	0.98	1.06	0.337
Sex (female)	1.13	0.59	2.16	0.710	1.92	0.66	5.55	0.229
Duration of asthma (per year)	1.01	0.99	1.04	0.429				
BMI (per kg/m^2^)	0.92	0.83	1.03	0.143				
History of exacerbation (presence)	3.90	1.95	7.80	<0.001	3.35	1.12	10.02	0.031
Rhinosinusitis (presence)	0.58	0.29	1.14	0.115	0.43	0.15	1.21	0.109
Family history of allergy disease (presence)	0.75	0.39	1.44	0.391				
Smoking (≥10 pack-years)	1.19	0.57	2.46	0.650				
FEV_1_ (per predicted %)	0.98	0.96	0.99	0.002	1.00	0.98	1.02	0.743
BDR (presence)	1.62	0.69	3.80	0.269				
Blood eosinophilia (per % of total WBC)	1.00	0.98	1.02	0.857				
Atopy (presence)	1.04	0.41	2.61	0.938				
Follow-up (maintained in the cohort)	0.64	0.33	1.21	0.166				
Log IgE	0.81	0.41	1.59	0.535				
ACT score variability	1.28	1.07	1.36	0.008	1.19	0.95	1.50	0.134
Inhaler compliance score	1.02	1.00	1.05	0.121	1.00	0.97	1.03	0.854

1 Adjusted for age, sex, and variables that had a p-value <0.1 in the unadjusted model.

Abbreviations: WBC, white blood cell; BMI, Body mass index.

**Table 5:** Multivariate analysis of risk factors for asthma-related healthcare use: Presence of hospitalizations.

Variable	Unadjusted				Adjusted^1^			
	OR	95% CI		p	OR	95% CI		p
Age (per year)	1.03	1.02	1.05	<0.001	1.03	1.01	1.06	0.021
Sex (female)	1.34	0.83	2.17	0.233	1.84	0.89	3.83	0.101
Duration of asthma (per year)	1.02	1.00	1.04	0.020				
BMI (per kg/m^2^)	1.02	0.95	1.09	0.683				
History of exacerbation (presence)	2.40	1.48	3.88	<0.001	2.01	0.99	4.10	0.055
Rhinosinusitis (presence)	0.67	0.40	1.12	0.124	0.64	0.31	1.31	0.222
Family history of allergy disease (presence)	1.01	0.63	1.62	0.978				
Smoking (≥10 pack-years)	1.09	0.63	1.88	0.77				
FEV_1_ (per predicted %)	0.98	0.97	0.99	0.0003	0.99	0.97	1.01	0.174
BDR (presence)	1.20	0.60	2.39	0.604				
Blood eosinophilia (per % of total WBC)	0.98	0.92	1.04	0.461				
Atopy (presence)	0.93	0.47	1.83	0.825				
Follow-up (maintained in the cohort)	1.05	0.64	1.73	0.843				
Log IgE	0.84	0.50	1.38	0.483				
ACT score variability	1.32	1.15	1.52	<0.001	1.31	1.11	1.55	0.002
Inhaler compliance score	1.02	1.00	1.03	0.062	1.00	0.99	1.02	0.723

1 Adjusted for age, sex, and variables that had a p-value <0.1 in the unadjusted model.

Abbreviations: WBC, white blood cell; BMI, Body mass index.

**Table 6:** Multivariate analysis of risk factors for asthma-related healthcare use: Per person cumulative duration (days) of systemic corticosteroid use.

Variable	Unadjusted				Adjusted^1^			
	β	95% CI		p	β	95% CI		p
Age (per year)	2.47	1.55	3.40	<0.001	1.04	−0.89	2.96	0.288
Sex (female)	−21.05	−49.60	7.49	0.148	27.15	−34.20	88.51	0.384
Duration of asthma (per year)	1.92	0.70	3.14	0.002	1.67	−0.63	3.97	0.155
BMI (per kg/m^2^)	−2.59	−6.76	1.58	0.222				
History of exacerbation (presence)	18.15	−10.43	46.73	0.213				
Rhinosinusitis (presence)	−35.67	−66.00	−5.34	0.021	8.81	−42.60	60.23	0.736
Family history of allergy disease (presence)	−9.57	−37.82	18.67	0.506				
Smoking (≥10 pack-years)	63.08	29.05	97.11	<0.001	1.26	−67.27	69.80	0.971
FEV_1_ (per predicted %)	−2.75	−3.35	−2.15	<0.001	−3.05	−4.23	−1.87	<0.001
BDR (presence)	75.74	32.28	119.20	0.001	37.00	−26.31	100.30	0.250
Blood eosinophilia (per % of total WBC)	−0.07	−0.32	0.17	0.554				
Atopy (presence)	−4.92	−42.87	33.04	0.799				
Follow-up (maintained in the cohort)	−1.94	−32.84	28.96	0.902				
Log IgE	−15.22	−49.97	19.53	0.389				
ACT score variability	7.71	−1.44	16.85	0.098	3.61	−8.86	16.08	0.568
Inhaler compliance score	0.52	−0.26	1.30	0.192				

1 Adjusted for age, sex, and variables that had a p-value <0.1 in the unadjusted model.

Abbreviations: WBC, white blood cell; BMI, Body mass index.

**Table 7:** Multivariate analysis of risk factors for asthma-related healthcare use: Presence of systemic corticosteroid use.

Variable	Unadjusted				Adjusted^1^			
	OR	95% CI		p	OR	95% CI		p
Age (per year)	1.02	1.01	1.03	0.005	1.01	0.99	1.02	0.370
Sex (female)	1.63	1.18	2.25	0.003	1.76	1.11	2.78	0.016
Duration of asthma (per year)	1.03	1.01	1.04	0.003	1.02	1.00	1.05	0.070
BMI (per kg/m^2^)	1.01	0.96	1.06	0.633				
History of exacerbation (presence)	2.12	1.49	3.01	<0.001	1.52	0.95	2.44	0.082
Rhinosinusitis (presence)	0.83	0.58	1.19	0.308				
Family history of allergy disease (presence)	1.15	0.84	1.58	0.391				
Smoking (≥10 pack-years)	0.88	0.60	1.29	0.520				
FEV_1_ (per predicted %)	0.99	0.98	1.00	0.004	0.98	0.97	0.99	0.040
BDR (presence)	1.00	0.57	1.73	0.987				
Blood eosinophilia (per % of total WBC)	1.00	0.99	1.02	0.682				
Atopy (presence)	0.71	0.47	1.07	0.099	0.75	0.42	1.36	0.347
Follow-up (maintained in the cohort)	2.14	1.54	2.98	<0.001	0.72	0.35	1.46	0.359
Log IgE	0.92	0.66	1.27	0.595				
ACT score variability	1.49	1.27	1.75	<0.001	1.41	1.20	1.65	<0.001
Inhaler compliance score	1.01	1.00	1.02	0.110				

1 Adjusted for age, sex, and variables that had a p-value <0.1 in the unadjusted model.

Abbreviations: WBC, white blood cell; BMI, Body mass index.

### Overall asthma-related healthcare use, Patterns of prescription of asthma medication

See Methods and Results S1 (Table S3 in File S1, Figure S1, Figure S2A, 2B, 2C, 2D, 2E, 2F).

## Discussion

In the present study, we analyzed patterns of and risk factors for healthcare use using data from a large-scaled asthma cohort and the Korean NHI claims database, which includes the almost entire Korean population. [15], [20] Although many studies have addressed the risk factors for asthma exacerbation, few reports have traced all asthma-related healthcare use because these studies only contained those who had been consistently followed by the researchers, excluding those patients who were lost to follow-up.

Definitely, we think that significant risk factors for every asthma-related healthcare use were assessed in real world through this reliable data analysis. The results indicate that different risk factors affect the different types of healthcare use among patients with asthma. In this study, increased age and high variability in ACT score were important determinants for hospital admissions, although previous history of asthma attack was associated with emergent department visits. While some results showed that age is directly proportional to asthma-related hospitalizations, [21], [22] multivariate analyses did not show that age is an independent risk factor for emergency department visits or hospitalizations, [23]–[27] indicating that asthma severity, as indicated by, for example, a low FEV_1_, and/or comorbidity, rather than chronological age, were more important factors. [27] In the current study, however, we revealed that old age could be an independent risk factor for asthma-related acute healthcare use regardless of asthma severity and comorbidity.

On the other hand, outpatient visits were affected by patients’ adherence to medical treatment. In addition, relatively severe asthma seemed to be related with frequent outpatient visits. Previous studies reported that poorly controlled asthma was highly associated with the number of outpatient visits. [28], [29] Long-standing asthma is a well-known risk factor of severe asthma with airway remodeling. [14], [30], [31] A low FEV_1_ and brittleness of asthma were also clinical criteria of severe/refectory asthma. [32], [33] Prescription of systemic corticosteroids may include both acute treatment for exacerbation and medication administered on an ‘as needed’ basis. It was associated with female gender, a low FEV_1_%, and high variability of ACT score in our study. Among these factors, however, only a low FEV_1_% was observed as a significant risk factor for longer cumulative duration of systemic corticosteroids therapy. Previous studies often used the prescription of systemic corticosteroids as an index of poor asthma control [34], [35], whereas there has been little studies using the duration of systemic corticosteroids therapy for measurement of healthcare utilization outcomes. According to our previous work, the number of prescribed days of systemic corticosteroids per person was higher in tertiary hospital than secondary or primary hospitals, suggesting that more severe asthma cases were included. [15] The result from present study therefore could indicate that the cumulative duration of systemic corticosteroids therapy more reliable index of healthcare use for severe asthma.

Another important finding is that although 81% (596/736) of patients maintained asthma-related outpatient visits in the third year, the prescription of ICS-based controllers was not adequately maintained in primary/secondary hospitals, which may be one of the major contributing factors to low adherence to ICS-based controllers. Proper maintenance of asthma controllers is important to prevent worsening of asthma. [18] However, only two studies examined long-term adherence to asthma drugs, and only in pediatric patients, finding that ICS use decreased to 46% after 1 year and 20% after 4 years, [36] and that 60–90% of asthma medication were stopped over the years [37].

In this study, we used a novel index of asthma, ‘ACT score variability’, in which higher values indicate a more brittle asthma. This index was highly correlated in this study with asthma-related healthcare use, such as outpatient visits, hospitalizations, and the presence of systemic corticosteroids, which demonstrates the relationship between asthma brittleness and asthma-related healthcare use. Because there is no good index for asthma brittleness, [38] the ACT score variability may be a useful marker of brittle asthma in future studies.

From analysis according to clinical phenotypes, patients with FAO, difficult-to-treat asthma, or who were in the severe obstructive asthma cluster had significantly higher healthcare use, reconfirming that previously identified indicators representing poor asthma control were significantly associated with higher healthcare use. [14], [15] In patients maintained in the cohort, the number of outpatient visits was higher, and the use of systemic corticosteroids was more frequent. When patients with FAO dropped out of the cohort, they tended to require more emergency visits and systemic corticosteroid use. Taken together, these results suggest that patients with higher asthma severity were more likely to remain in tertiary hospitals and that attending tertiary hospitals might be more beneficial for asthma management in patients with FAO.

This study has two limitations. First, some outcome variables, such as frequency of outpatient visits and systemic corticosteroid use, might not be directly associated with poor asthma control, since systemic corticosteroids could be prescribed for possible future exacerbations. Second, our asthma cohort could not represent general adult asthma patients in Korea, although it was composed of all severities of asthma according to test results and the opinions of experts. In addition, the caution may be needed to interpret the data which sample sizes were small in many stratified analyses.

In conclusion, here we present a novel approach to asthma research that involved using both an asthma cohort and a national insurance claims database. We determined important risk factors for increased asthma-related healthcare use. Long-term adherence to ICS-based controllers in Korean adults was lower than expected. More attention to and education about patients with risk factors for increased healthcare use are required to reduce asthma-related healthcare use.

## Supporting Information

Figure S1 **The distribution of patients according to asthma-related healthcare use during the index period.** (A) The distribution of patients according to the number of outpatient visits. (B) The distribution of patients according to the number of hospitalizations (C) The distribution of patients according to the number of emergency department visits.
(TIF)

Figure S2 **Patterns of prescription for asthma medications during the index period.** Graphs show the proportion of patients prescribed each class of medication at least once by healthcare institution. The total number of patients (100%) was 736. (A) ICS/LABA (B) ICS (C) Oral leukotriene antagonists (D) Theophylline derivatives (E) SABA (F) Systemic corticosteroids.
(TIF)

File S1 **Supporting tables. Table S1** Characteristics of the four clusters of COREA patients (adapted from the reference 16). **Table S2** Demographic data and asthma-related healthcare use in Korea: NHI claims data (adapted from the reference 15). **Table S3** Overall asthma-related healthcare use during the index period. **Table S4** Asthma-related healthcare use according to baseline clinical characteristics. **Table S5** Asthma-related healthcare use according to clinical phenotypes. **Table S6** Asthma-related healthcare use according to adherence to the cohort.
(DOCX)

Methods and Results S1 **Methods S1.** SUPPLEMENT METHODS. **Results S1.** SUPPLEMENT RESULTS.
(DOCX)
