# Supplementary figures and images for: Risk Factors for Asthma-Related Healthcare Use: Longitudinal Analysis Using the NHI Claims Database in a Korean Asthma Cohort
Source: PLoS One. 2014 Nov 14;9(11):e112844. doi: 10.1371/journal.pone.0112844 (PMC4232512; doi:10.1371/journal.pone.0112844)

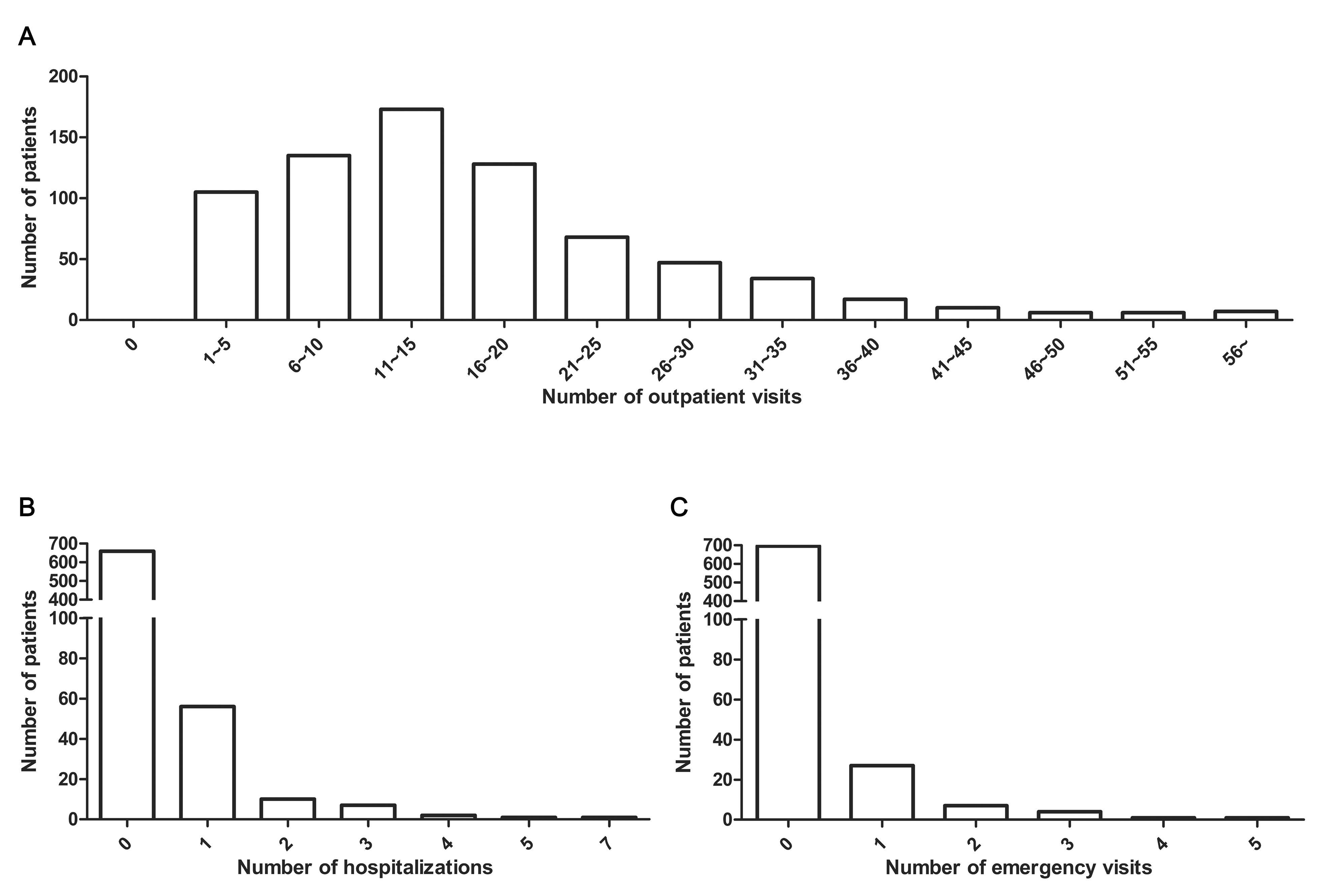

Supplement: Figure S1 — The distribution of patients according to asthma-related healthcare use during the index period. (A) The distribution of patients according to the number of outpatient visits. (B) The distribution of patients according to the number of hospitalizations (C) The distribution of patients according to the number of emergency department visits. (TIF) [file pone.0112844.s001.tif]

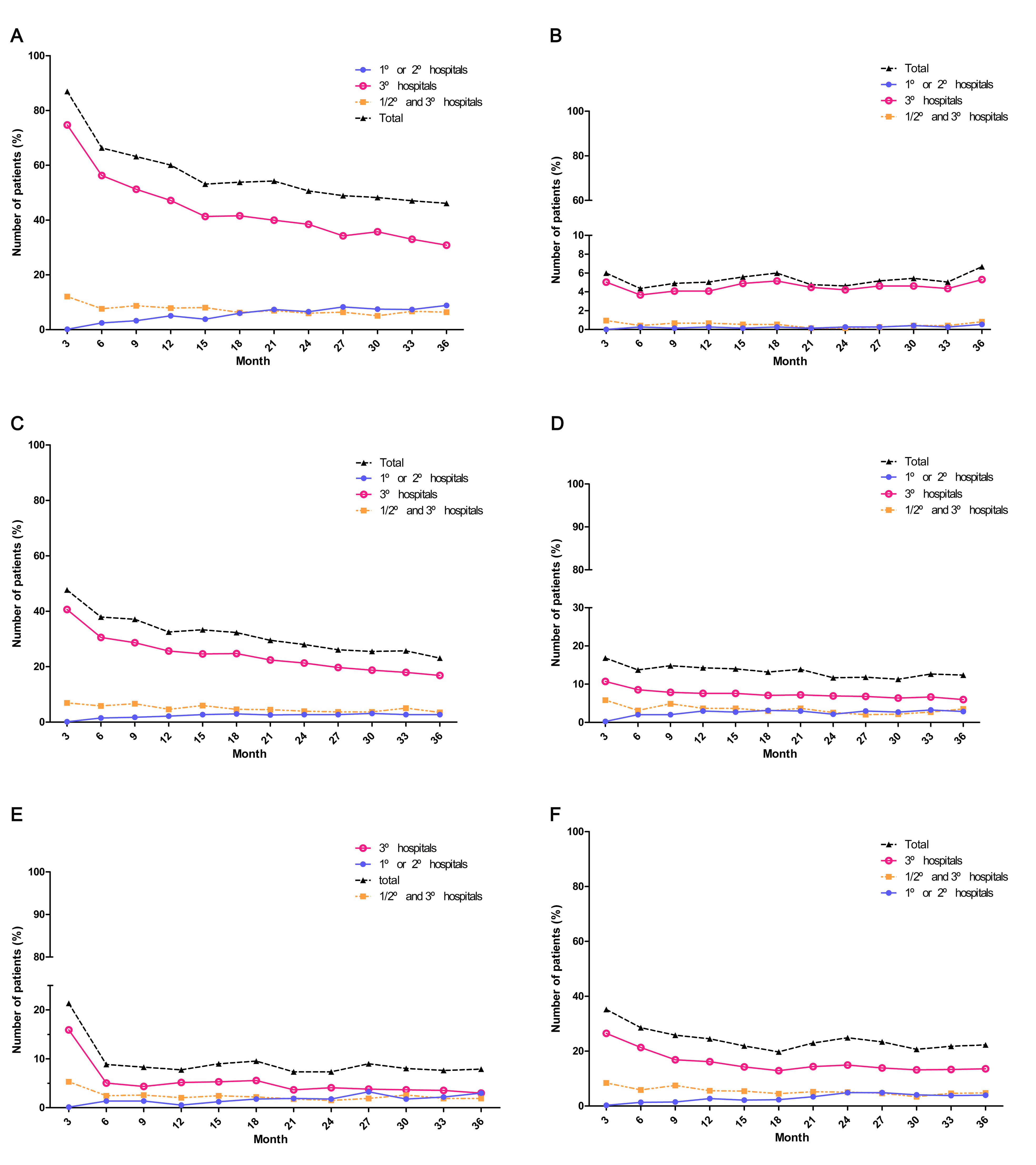

Supplement: Figure S2 — Patterns of prescription for asthma medications during the index period. Graphs show the proportion of patients prescribed each class of medication at least once by healthcare institution. The total number of patients (100%) was 736. (A) ICS/LABA (B) ICS (C) Oral leukotriene antagonists (D) Theophylline derivatives (E) SABA (F) Systemic corticosteroids. (TIF) [file pone.0112844.s002.tif]
