# Supplementary material for: Risk Factors for Asthma-Related Healthcare Use: Longitudinal Analysis Using the NHI Claims Database in a Korean Asthma Cohort
Source: PLoS One. 2014 Nov 14;9(11):e112844. doi: 10.1371/journal.pone.0112844 (PMC4232512; doi:10.1371/journal.pone.0112844)
Supplement: File S1 — Supporting tables. Table S1 Characteristics of the four clusters of COREA patients (adapted from the reference 16). Table S2 Demographic data and asthma-related healthcare use in Korea: NHI claims data (adapted from the reference 15). Table S3 Overall asthma-related healthcare use during the index period. Table S4 Asthma-related healthcare use according to baseline clinical characteristics. Table S5 Asthma-related healthcare use according to clinical phenotypes. Table S6 Asthma-related healthcare use according to adherence to the cohort. (DOCX) [file pone.0112844.s003.docx]

Table S1 Characteristics of the four clusters of COREA patients (adapted from the reference 16)

|  | **Cluster A**  **(n=81)** | **Cluster B**  **(n=151)** | **Cluster C**  **(n=253)** | **Cluster D**  **(n=239)** | **P value** |
| --- | --- | --- | --- | --- | --- |
| Gender (% of male) | 97.4 | 41.3 | 47.2 | 25.8 | <0.001 |
| Age at onset, yr | 46.2±12.6 | 38.8±15.1 | 21.8±9.4 | 47.8±9.7 | <0.001 |
| Body mass index (kg/m^2^) | 24.71±2.43 | 23.50±3.09 | 23.09±3.45 | 24.35 ± 3.07 | <0.001 |
| Smoking (pack-years) | 34.4±14.1 | 3.2±6.5 | 2.0±3.7‡ | 2.0± 5.3 | <0.001 |
| Atopy, % positive | 34.6 | 49.7 | 66.0 | 53.6 | <0.001 |
| History of HU (% positive) | 22.2 | 46.4 | 36.8 | 23.4 | <0.001 |
| Rhinitis (% of positivity) | 58.3 | 65.3 | 73.4 | 65.0 | 0.056 |
| PostBD FEV1 (%) | 82.47±16.45 | 56.48±13.7 | 88.62±11.24 | 97.94±11.5 | <0.001 |
| PostBD % increased of FEV1 | 5.90±7.75 | 12.00±12.07 | 6.24±7.52 | 4.34±6.56 | <0.001 |
| PostBD FEV1/FVC (%) | 71.44±11.76 | 67.84±12.40 | 84.76±8.69 | 80.25±7.40 | <0.001 |
| PC20 (mg/ml) | 4.95±6.14 | 3.11±5.35 | 5.49±6.04 | 5.64±6.47 | 0.03 |
| Blood eosinophils (%) | 4.80± 4.33 | 5.60± 5.48 | 5.50± 6.43 | 5.02± 4.70 | 0.612 |
| Blood eosinophil count (/mm^3^) | 388.4±376.6 | 409.6±454.2 | 377.9±354.3 | 331.0±301.1 | 0.247 |
| Log blood eosinophil count | 2.42±0.44 | 2.40±0.47 | 2.41±0.41 | 2.34±0.42 | 0.445 |
| Blood neutrophil count (/mm^3^) | 4397± 1971 | 4672± 3168 | 4107± 1775 | 3631± 1606 | <0.001 |
| Serum CRP (mg/dl) | 0.78± 2.43 | 0.61± 0.90 | 0.21± 0.35 | 0.37± 0.96 | 0.02 |
| Serum uric acid levels (mg/dl) | 5.85± 1.35 | 5.13± 1.51 | 5.10± 1.39 | 4.85± 1.60 | <0.001 |
| Serum Total IgE (Log IU/L) | 2.41±0.62 | 2.26±0.59 | 2.35±0.60 | 2.14± 0.61 | 0.008 |

Comparison between study groups is based on One-way ANOVA analysis for continuous variables and a χ^2^ test for proportions. Data indicate means ± SDs.

Table S2 Demographic data and asthma-related healthcare use in Korea: NHI claims data (adapted from the reference 15)

|  | | **2006** | | **2007** | | **2008** | | **2009** | | **2010** | |
| --- | --- | --- | --- | --- | --- | --- | --- | --- | --- | --- | --- |
|  |  | n | % | n | % | n | % | n | % | n | % |
| **Total N. of population included in the NHI database (Age ≥ 18)** | | 38,288,695 |  | 38,849,158 |  | 39,349,430 |  | 39,889,189 |  | 40,435,086 |  |
|  | Male | 18,974,476 |  | 19,258,584 |  | 19,518,883 |  | 19,795,167 |  | 20,075,632 |  |
|  | Female | 19,314,219 |  | 19,590,571 |  | 19,830,547 |  | 20,094,022 |  | 20,359,454 |  |
| **Total subjects** | | 1,892,953 | 100% | 1,956,491 | 100% | 2,037,322 | 100% | 2,160,255 | 100% | 2,307,581 | 100% |
| **Gender** | |  |  |  |  |  |  |  |  |  |  |
|  | Male | 713,146 | 37.7% | 744,034 | 38.0% | 779,733 | 38.3% | 824,460 | 38.2% | 892,309 | 38.7% |
|  | Female | 1,179,807 | 62.3% | 1,212,457 | 62.0% | 1,257,589 | 61.7% | 1,335,795 | 61.8% | 1,415,272 | 61.3% |
| **Age** (mean ± SD) | | 51.69 ± 18.70 | | 51.82 ± 20.30 | | 52.86 ± 30.33 | | 51.95 ± 19.94 | | 51.81 ± 19.52 | |
| **Insurance type** | |  | |  | |  | |  | |  | |
|  | Health Insurance | 1,755,826 | 92.8% | 1,817,128 | 92.9% | 1,892,781 | 92.9% | 2,014,674 | 93.3% | 2,162,462 | 93.7% |
|  | Medical Aid | 137,127 | 7.2% | 139,363 | 7.1% | 144,541 | 7.1% | 145,581 | 6.7% | 145,119 | 6.3% |
| **Outpatient visits** | |  | |  | |  | |  | |  | |
|  | No | 35,585 | 1.9% | 40,373 | 2.1% | 46,058 | 2.3% | 47,632 | 2.2% | 51,558 | 2.2% |
|  | Yes | 1,857,368 | 98.1% | 1,916,118 | 97.9% | 1,991,264 | 97.7% | 2,112,623 | 97.8% | 2,256,023 | 97.8% |
|  | (Mean ± SD)^#^ | 4.82 ± 8.02 | | 4.17 ± 6.71 | | 3.62 ± 5.90 | | 3.55 ± 5.73 | | 3.44 ± 5.50 | |
| **Hospitalizations** | |  | |  | |  | |  | |  | |
|  | No | 1,838,326 | 97.1% | 1,896,721 | 96.9% | 1,971,858 | 96.8% | 2,094,510 | 97.0% | 2,237,816 | 97.0% |
|  | Yes | 54,627 | 2.9% | 59,770 | 3.1% | 65,464 | 3.2% | 65,745 | 3.0% | 69,765 | 3.0% |
|  | (Mean ± SD)^#^ | 1.6 ± 1.38 | | 1.62 ± 1.44 | | 1.57 ± 1.3 | | 1.57 ± 1.34 | | 1.57 ± 1.32 | |
| **Hospitalization days** (Mean ± SD)^#^ | |  | |  | |  | |  | |  | |
|  | per person per year | 21.36 ± 31.04 | | 21.71 ± 32.76 | | 20.42 ± 27.75 | | 19.66 ± 26.95 | | 19.26 ± 25.90 | |
|  | per each admission | 13.34 ± 20.75 | | 13.38 ± 21.92 | | 13.03 ± 18.45 | | 12.54 ± 17.98 | | 12.30 ± 17.13 | |
| **ICU hospitalizations** | |  | |  | |  | |  | |  | |
|  | No | 1,884,200 | 99.54% | 1,947,100 | 99.52% | 2,027,305 | 99.51% | 2,150,661 | 99.56% | 2,297,479 | 99.56% |
|  | Yes | 8,753 | 0.46% | 9,391 | 0.48% | 10,017 | 0.49% | 9,594 | 0.44% | 10,102 | 0.44% |
|  | (Mean±SD)^#^ | 1.00 ± 0.0 | | 1.00 ± 0.0 | | 1.00 ± 0.0 | | 1.00 ± 0.0 | | 1.00 ± 0.0 | |
| **Emergency dept. visits*** | |  | |  | |  | |  | |  | |
|  | No | 1,850,196 | 97.7% | 1,825,258 | 97.6% | 1,988,450 | 97.6% | 2,109,073 | 97.6% | 2,253,988 | 97.7% |
|  | Yes | 42,757 | 2.3% | 45,068 | 2.4% | 48,872 | 2.4% | 51,182 | 2.4% | 53,593 | 2.3% |
|  | (Mean±SD)^#^ | 1.43 ± 1.12 | | 1.48 ± 0.95 | | 1.51± 0.81 | | 1.43 ± 1.41 | | 1.47 ± 1.06 | |
| **Pulmonary function tests** | |  | |  | |  | |  | |  | |
|  | No | 1,685,933 | 89.1% | 1,737,171 | 88.8% | 1,815,571 | 89.1% | 1,926,474 | 89.2% | 2,077,226 | 90.0% |
|  | Yes | 207,020 | 10.9% | 219,320 | 11.2% | 221,751 | 10.9% | 233,781 | 10.8% | 230,355 | 10.0% |
|  | (Mean±SD)^#^ | 1.28 ± 0.99 | | 1.28 ± 0.99 | | 1.28 ± 0.99 | | 1.27 ± 0.91 | | 1.27 ± 0.89 | |

* the number of patients who have been treated in an emergency department due to asthma exacerbation without admission at least once

^#^ the mean values of subjects who experienced at least one event

Table S3 Overall asthma-related healthcare use during the index period

| **Patterns of usage** | **Index period** | **Number  of patients** | **Total number of visits** | **Per person visits (mean ± SD)** | **Number of visits by  the type of medical institution** | | | |
| --- | --- | --- | --- | --- | --- | --- | --- | --- |
|  |  |  |  |  |  |  |  |  |
|  |  |  |  |  | **Primary and secondary** | | **Tertiary** | |
| Outpatient visits | 1^st^ year | 725 | 4,686 | 6.5 ± 3.6 | 829 | 17.7% | 3,857 | 82.3% |
|  | 2^nd^ year | 633 | 3,746 | 5.9 ± 4.4 | 1,234 | 32.9% | 2,512 | 67.1% |
|  | 3^rd^ year | 596 | 3,575 | 6.0 ± 4.6 | 1,381 | 38.6% | 2,194 | 61.4% |
|  | Total | 736 | 12,007 | 16.3 ± 11.3 | 3,444 | 28.7% | 8,563 | 71.3% |
| Hospitalizations | 1^st^ year | 34 | 50 | 1.5 ± 1.0 | 1 | 2.0% | 49 | 98.0% |
|  | 2^nd^ year | 32 | 33 | 1.1 ± 0.2 | 2 | 6.1% | 31 | 93.9% |
|  | 3^rd^ year | 28 | 33 | 1.2 ± 0.4 | 6 | 18.2% | 27 | 81.8% |
|  | Total | 77 | 116 | 1.5 ± 1.0 | 9 | 7.8% | 107 | 92.2% |
| Emergency department visits | 1^st^ year | 17 | 26 | 1.5 ± 1.0 | 0 | 0.0% | 26 | 100.0% |
|  | 2^nd^ year | 12 | 14 | 1.2 ± 0.6 | 0 | 0.0% | 14 | 100.0% |
|  | 3^rd^ year | 21 | 23 | 1.1 ± 0.3 | 2 | 8.7% | 40 | 91.3% |
|  | Total | 40 | 63 | 1.6 ± 1.1 | 2 | 3.2% | 61 | 96.8% |

Table S4 Asthma-related healthcare use according to baseline clinical characteristics

| **Variable** | **Total number  of  patients  (n = 736)** | **Outpatient visits** | | | | **Emergency department visits** | | | | | | **Hospitalizations** | | | | | | | | | **The use of systemic corticosteroids** | | | | | |
| --- | --- | --- | --- | --- | --- | --- | --- | --- | --- | --- | --- | --- | --- | --- | --- | --- | --- | --- | --- | --- | --- | --- | --- | --- | --- | --- |
|  |  | **Number  of  patients** | **Per capita frequency of outpatient visits** | | | **Number  of  patients** | **Per capita frequency of emergency department visits** | | | **Presence of  emergency department visits** | | **Number  of  patients** | **Per capita frequency of hospitalizations** | | | **Per capita cumulative duration (days) of hospitalizations** | | | **Presence of hospitalizations** | | **Number  of  patients** | **Per capita cumulative duration (days) of systemic steroids use** | | | **Presence of systemic steroids use** | |
|  |  |  | **mean** | **SD** | **p** |  | **mean** | **SD** | **p** | **percent** | **p** |  | **mean** | **SD** | **p** | **mean** | **SD** | **p** | **percent** | **p** |  | **mean** | **SD** | **p** | **percent** | **p** |
| Age (years) |  |  |  |  | <0.001 |  |  |  | 0.341 |  | 0.226 |  |  |  | 0.245 |  |  | 0.912 |  | 0.001 |  |  |  | 0.001 |  | 0.016 |
| <30 | 97 | 97 | 12.5 | 9.9 |  | 6 | 2.2 | 2.0 |  | 6.2% |  | 7 | 2.0 | 1.8 |  | 12.9 | 14.8 |  | 7% |  | 57 | 40.3 | 87.9 |  | 59% |  |
| ≥30 and <60 | 407 | 407 | 16.0 | 11.5 |  | 17 | 1.5 | 0.7 |  | 4.2% |  | 31 | 1.3 | 0.7 |  | 17.6 | 38.1 |  | 8% |  | 297 | 77.3 | 149.8 |  | 73% |  |
| ≥60 | 232 | 232 | 18.5 | 11.1 |  | 17 | 1.5 | 0.9 |  | 7.3% |  | 39 | 1.6 | 1.0 |  | 17.4 | 16.1 |  | 17% |  | 169 | 121.8 | 198.6 |  | 73% |  |
| Sex |  |  |  |  | 0.005 |  |  |  | 0.947 |  | 0.707 |  |  |  | 0.146 |  |  | 0.989 |  | 0.232 |  |  |  | 0.159 |  | 0.003 |
| Male | 334 | 334 | 15.1 | 10.5 |  | 17 | 1.6 | 1.3 |  | 5.1% |  | 30 | 1.7 | 1.2 |  | 17.1 | 15.3 |  | 9% |  | 219 | 99.9 | 178.9 |  | 66% |  |
| Female | 402 | 402 | 17.4 | 11.9 |  | 23 | 1.6 | 0.8 |  | 5.7% |  | 47 | 1.4 | 0.8 |  | 17.0 | 32.4 |  | 12% |  | 304 | 78.8 | 152.3 |  | 76% |  |
| Atopy |  |  |  |  | 0.022 |  |  |  | 0.553 |  | 0.938 |  |  |  | 0.697 |  |  | 0.121 |  | 0.825 |  |  |  | 0.749 |  | 0.099 |
| Presence | 220 | 220 | 13.7 | 9.3 |  | 10 | 1.3 | 0.7 |  | 4.6% |  | 18 | 1.3 | 0.6 |  | 10.3 | 9.0 |  | 8% |  | 139 | 63.7 | 124.5 |  | 63% |  |
| Absence | 205 | 205 | 16.0 | 11.0 |  | 9 | 1.6 | 1.1 |  | 4.4% |  | 18 | 1.4 | 1.0 |  | 18.4 | 19.4 |  | 9% |  | 145 | 68.6 | 133.4 |  | 71% |  |
| BMI (kg/m^2^) |  |  |  |  | 0.716 |  |  |  | 0.843 |  | 0.644 |  |  |  | 0.185 |  |  | 0.394 |  | 0.545 |  |  |  | 0.311 |  | 0.274 |
| <25 | 455 | 455 | 16.5 | 11.1 |  | 27 | 1.5 | 0.8 |  | 5.9% |  | 43 | 1.6 | 1.0 |  | 20.2 | 34.6 |  | 9% |  | 322 | 97.0 | 177.5 |  | 71% |  |
| ≥25 and <30 | 231 | 231 | 16.1 | 11.9 |  | 11 | 1.5 | 0.7 |  | 4.8% |  | 28 | 1.2 | 0.5 |  | 12.5 | 9.1 |  | 12% |  | 161 | 76.4 | 149.1 |  | 70% |  |
| ≥30 | 35 | 35 | 14.9 | 9.6 |  | 1 | 1.0 | . |  | 2.9% |  | 4 | 1.5 | 1.0 |  | 7.0 | 8.8 |  | 11% |  | 29 | 64.1 | 95.8 |  | 83% |  |
| Rhinosinusitis |  |  |  |  | 0.004 |  |  |  | 0.358 |  | 0.111 |  |  |  | 0.036 |  |  | 0.138 |  | 0.122 |  |  |  | 0.034 |  | 0.308 |
| Presence | 454 | 454 | 15.3 | 10.8 |  | 19 | 1.3 | 0.7 |  | 4.2% |  | 39 | 1.2 | 0.5 |  | 11.4 | 10.9 |  | 9% |  | 319 | 74.4 | 145.5 |  | 70% |  |
| Absence | 227 | 227 | 17.9 | 11.7 |  | 16 | 1.6 | 0.9 |  | 7.1% |  | 28 | 1.7 | 1.2 |  | 23.4 | 40.4 |  | 12% |  | 168 | 110.1 | 189.3 |  | 74% |  |
| Presence of family history of allergic diseases |  |  |  |  | 0.630 |  |  |  | 0.028 |  | 0.389 |  |  |  | 0.054 |  |  | 0.140 |  | 0.978 |  |  |  | 0.506 |  | 0.391 |
| Presence | 343 | 343 | 16.1 | 11.5 |  | 16 | 1.2 | 0.4 |  | 4.7% |  | 36 | 1.3 | 0.7 |  | 12.4 | 13.5 |  | 11% |  | 249 | 82.6 | 154.7 |  | 73% |  |
| Absence | 393 | 393 | 16.5 | 11.2 |  | 24 | 1.8 | 1.3 |  | 6.1% |  | 41 | 1.7 | 1.2 |  | 21.1 | 34.3 |  | 10% |  | 274 | 92.2 | 172.4 |  | 70% |  |
| Duration of asthma (years) |  |  |  |  | 0.006 |  |  |  | 0.133 |  | 0.292 |  |  |  | 0.277 |  |  | 0.568 |  | 0.007 |  |  |  | <0.001 |  | 0.013 |
| <10 | 435 | 435 | 15.5 | 11.1 |  | 21 | 1.8 | 1.3 |  | 4.8% |  | 35 | 1.7 | 1.3 |  | 19.3 | 37.3 |  | 8% |  | 294 | 63.9 | 130.3 |  | 68% |  |
| ≥10 | 285 | 285 | 17.8 | 11.5 |  | 19 | 1.3 | 0.6 |  | 6.7% |  | 41 | 1.4 | 0.7 |  | 15.5 | 13.4 |  | 14% |  | 217 | 122.3 | 199.1 |  | 76% |  |
| Smoking |  |  |  |  | 0.026 |  |  |  | 0.247 |  | 0.764 |  |  |  | 0.049 |  |  | 0.764 |  | 0.315 |  |  |  | 0.264 |  | 0.007 |
| Current | 103 | 103 | 13.9 | 10.8 |  | 6 | 1.5 | 0.8 |  | 5.8% |  | 11 | 1.4 | 0.8 |  | 11.7 | 7.7 |  | 11% |  | 60 | 84.9 | 183.7 |  | 58% |  |
| Ex- | 328 | 328 | 16.1 | 10.6 |  | 15 | 1.9 | 1.5 |  | 4.6% |  | 28 | 1.9 | 1.3 |  | 18.8 | 17.9 |  | 9% |  | 236 | 100.1 | 175.0 |  | 72% |  |
| Non- | 294 | 294 | 17.4 | 12.2 |  | 17 | 1.2 | 0.4 |  | 5.8% |  | 36 | 1.3 | 0.6 |  | 17.7 | 35.9 |  | 12% |  | 219 | 74.9 | 147.2 |  | 74% |  |
| Pack-years of smoking |  |  |  |  | 0.865 |  |  |  | 0.790 |  | 0.650 |  |  |  | 0.418 |  |  | 0.984 |  | 0.770 |  |  |  | 0.002 |  | 0.520 |
| <10 and non-smokers | 390 | 390 | 16.4 | 11.9 |  | 21 | 1.4 | 1.1 |  | 5.4% |  | 42 | 1.4 | 1.0 |  | 17.7 | 33.6 |  | 11% |  | 283 | 69.4 | 137.1 |  | 73% |  |
| ≥10 | 190 | 190 | 16.5 | 10.7 |  | 12 | 1.3 | 0.7 |  | 6.3% |  | 22 | 1.6 | 1.0 |  | 17.5 | 15.5 |  | 12% |  | 133 | 132.4 | 211.9 |  | 70% |  |
| History of exacerbation |  |  |  |  | <0.001 |  |  |  | 0.543 |  | <0.0001 |  |  |  | 0.338 |  |  | 0.892 |  | <0.001 |  |  |  | 0.213 |  | <0.001 |
| Presence | 286 | 286 | 18.5 | 11.8 |  | 28 | 1.6 | 1.1 |  | 9.8% |  | 45 | 1.6 | 1.1 |  | 16.7 | 32.8 |  | 16% |  | 229 | 98.1 | 166.9 |  | 80% |  |
| Absence | 443 | 443 | 15.0 | 10.8 |  | 12 | 1.4 | 1.0 |  | 2.7% |  | 32 | 1.4 | 0.9 |  | 17.5 | 15.5 |  | 7% |  | 290 | 79.9 | 162.7 |  | 65% |  |
| Blood eosinophils (% of total WBC) |  |  |  |  | 0.391 |  |  |  | 0.825 |  | 0.794 |  |  |  | 0.603 |  |  | 0.109 |  | 0.098 |  |  |  | 0.046 |  | 0.292 |
| <5 | 367 | 367 | 17.0 | 11.7 |  | 22 | 1.6 | 0.9 |  | 6.0% |  | 45 | 1.5 | 0.9 |  | 20.0 | 33.7 |  | 12% |  | 250 | 111.4 | 199.3 |  | 68% |  |
| ≥5 | 237 | 237 | 16.1 | 11.0 |  | 13 | 1.7 | 1.5 |  | 5.5% |  | 19 | 1.4 | 1.2 |  | 10.9 | 10.1 |  | 8% |  | 171 | 78.5 | 137.9 |  | 72% |  |
| Sputum eosinophils (% of total WBC) |  |  |  |  | 0.918 |  |  |  | 0.566 |  | 0.937 |  |  |  | 0.550 |  |  | 0.354 |  | 0.429 |  |  |  | 0.647 |  | 0.961 |
| <3 | 38 | 38 | 15.2 | 13.7 |  | 2 | 2.5 | 2.1 |  | 5.3% |  | 5 | 1.8 | 1.8 |  | 26.4 | 28.3 |  | 13% |  | 26 | 125.1 | 218.5 |  | 68% |  |
| ≥3 | 125 | 125 | 15.5 | 11.8 |  | 7 | 1.3 | 0.5 |  | 5.6% |  | 11 | 1.3 | 0.5 |  | 12.8 | 12.7 |  | 9% |  | 85 | 148.3 | 227.4 |  | 68% |  |
| BDR |  |  |  |  | 0.148 |  |  |  | 0.627 |  | 0.265 |  |  |  | 0.759 |  |  | 0.221 |  | 0.604 |  |  |  | 0.008 |  | 0.987 |
| Positive | 93 | 93 | 19.5 | 12.7 |  | 8 | 1.6 | 0.9 |  | 8.6% |  | 12 | 1.5 | 0.8 |  | 13.0 | 9.5 |  | 13% |  | 73 | 159.4 | 226.8 |  | 78% |  |
| Negative | 364 | 364 | 17.4 | 11.9 |  | 20 | 1.5 | 0.8 |  | 5.5% |  | 40 | 1.6 | 1.0 |  | 20.7 | 35.3 |  | 11% |  | 286 | 83.6 | 150.3 |  | 79% |  |
| Initial FEV_1_/FVC (%) |  |  |  |  | 0.004 |  |  |  | 0.433 |  | 0.067 |  |  |  | 0.105 |  |  | 0.025 |  | 0.123 |  |  |  | <0.001 |  | 0.297 |
| <70 | 218 | 218 | 18.3 | 12.3 |  | 17 | 1.6 | 0.9 |  | 7.8% |  | 29 | 1.7 | 1.1 |  | 27.9 | 40.6 |  | 13% |  | 159 | 141.2 | 213.7 |  | 73% |  |
| ≥70 | 478 | 478 | 15.5 | 10.7 |  | 21 | 1.4 | 0.7 |  | 4.4% |  | 45 | 1.3 | 0.7 |  | 10.0 | 7.0 |  | 9% |  | 330 | 64.6 | 127.3 |  | 69% |  |
| Initial FEV_1_ (predicted %) |  |  |  |  | <0.001 |  |  |  | 0.931 |  | 0.013 |  |  |  | 0.435 |  |  | 0.282 |  | 0.001 |  |  |  | <0.001 |  | 0.013 |
| <60 | 106 | 106 | 21.6 | 12.7 |  | 12 | 1.5 | 0.7 |  | 11.3% |  | 22 | 1.6 | 1.0 |  | 24.8 | 45.2 |  | 21% |  | 87 | 217.9 | 256.8 |  | 82% |  |
| ≥60 and <80 | 186 | 186 | 17.2 | 11.4 |  | 8 | 1.4 | 0.7 |  | 4.8% |  | 19 | 1.4 | 0.8 |  | 15.2 | 13.3 |  | 10% |  | 128 | 94.7 | 157.8 |  | 69% |  |
| ≥80 | 397 | 397 | 14.6 | 10.5 |  | 17 | 1.4 | 0.9 |  | 4.3% |  | 31 | 1.3 | 0.8 |  | 12.9 | 13.8 |  | 8% |  | 268 | 46.5 | 94.7 |  | 68% |  |
| Initial severity of asthma |  |  |  |  | <0.001 |  |  |  | 0.838 |  | 0.020 |  |  |  | 0.331 |  |  | 0.724 |  | 0.004 |  |  |  | <0.001 |  | 0.104 |
| Mild intermittent | 32 | 32 | 11.3 | 9.5 |  | 2 | 1.0 | 0.0 |  | 6.3% |  | 2 | 1.0 | 0.0 |  | 17.5 | 10.6 |  | 6% |  | 22 | 49.6 | 62.1 |  | 69% |  |
| Mild persistent | 132 | 132 | 15.6 | 12.0 |  | 2 | 1.5 | 0.7 |  | 1.5% |  | 7 | 1.4 | 0.8 |  | 18.0 | 15.8 |  | 5% |  | 83 | 72.1 | 132.9 |  | 63% |  |
| Moderate persistent | 321 | 321 | 14.8 | 9.3 |  | 14 | 1.4 | 0.7 |  | 4.4% |  | 28 | 1.3 | 0.6 |  | 12.1 | 11.2 |  | 8,72% |  | 234 | 58.9 | 120.5 |  | 73% |  |
| Severe persistent | 201 | 201 | 20.0 | 12.6 |  | 18 | 1.5 | 0.9 |  | 9.0% |  | 33 | 1.6 | 1.1 |  | 20.2 | 37.5 |  | 16% |  | 150 | 146.1 | 225.0 |  | 75% |  |

Abbreviations: SD, standard deviation; WBC, white blood cell

P-values were determined with a chi-square test, Student's *t*-test, or ANOVA.

Table S5 Asthma-related healthcare use according to clinical phenotypes

| **Variable** | **Total number  of  patients  (n = 736)** | **Outpatient visits** | | | | **Emergency department visits** | | | | | | **Hospitalizations** | | | | | | | | | **The use of systemic corticosteroids** | | | | | |
| --- | --- | --- | --- | --- | --- | --- | --- | --- | --- | --- | --- | --- | --- | --- | --- | --- | --- | --- | --- | --- | --- | --- | --- | --- | --- | --- |
|  |  | **Number  of  patients** | **Per capita frequency of outpatient visits** | | | **Number  of  patients** | **Per capita frequency of emergency department visits** | | | **Presence of  emergency department visits** | | **Number  of  patients** | **Per capita frequency of hospitalizations** | | | **Per capita cumulative duration (days) of hospitalizations** | | | **Presence of hospitalizations** | | **Number  of  patients** | **Per capita cumulative duration (days) of systemic steroids use** | | | **Presence of systemic steroids use** | |
|  |  |  | **mean** | **SD** | **p** |  | **mean** | **SD** | **p** | **percent** | **p** |  | **mean** | **SD** | **p** | **mean** | **SD** | **p** | **percent** | **p** |  | **mean** | **SD** | **p** | **percent** | **p** |
| Airway reversibility |  |  |  |  | 0.002 |  |  |  | 0.315 |  | 0.125 |  |  |  | 0.452 |  |  | 0.099 |  | 0.150 |  |  |  | 0.002 |  | 0.088 |
| FAO | 91 | 91 | 19.6 | 9.9 |  | 7 | 1.9 | 0.9 |  | 7.69% |  | 12 | 1.6 | 0.9 |  | 19.5 | 17.4 |  | 13.19% |  | 72 | 162.4 | 237.8 |  | 79.12% |  |
| RAO | 565 | 565 | 15.8 | 10.8 |  | 23 | 1.5 | 0.8 |  | 4.07% |  | 48 | 1.4 | 0.8 |  | 12.2 | 12.5 |  | 8.50% |  | 398 | 69.3 | 135.8 |  | 70.44% |  |
| Treatability |  |  |  |  | <0.001 |  |  |  | 0.244 |  | 0.124 |  |  |  | 0.076 |  |  | 0.742 |  | 0.011 |  |  |  | 0.001 |  | 0.001 |
| Non-difficult-to-treat asthma | 314 | 314 | 17.6 | 9.3 |  | 12 | 1.6 | 0.8 |  | 3.82% |  | 28 | 1.3 | 0.6 |  | 15.5 | 15.2 |  | 8.92% |  | 234 | 71.4 | 128.8 |  | 74.52% |  |
| Difficult-to-treat asthma | 60 | 60 | 23.0 | 10.4 |  | 5 | 2.2 | 1.3 |  | 8.33% |  | 12 | 2.1 | 1.4 |  | 17.3 | 16.8 |  | 20.00% |  | 56 | 195.5 | 254.8 |  | 93.33% |  |
| Variability of ACT score (mean ± SD = 2.3 ± 1.7) |  |  |  |  | <0.001 |  |  |  | 0.751 |  | 0.100 |  |  |  | 0.531 |  |  | 0.269 |  | 0.022 |  |  |  | 0.012 |  | 0.424 |
| <2 | 258 | 258 | 15.1 | 8.2 |  | 8 | 1.5 | 0.8 |  | 3.10% |  | 18 | 1.4 | 0.8 |  | 12.2 | 9.3 |  | 6.98% |  | 167 | 65.8 | 140.1 |  | 64.73% |  |
| ≥2 | 261 | 261 | 20.3 | 10.5 |  | 16 | 1.6 | 1.0 |  | 6.13% |  | 34 | 1.6 | 1.0 |  | 16.2 | 16.4 |  | 13.03% |  | 227 | 105.7 | 171.6 |  | 86.97% |  |
| Compliance of all drugs (mean ± SD = 87.1 ± 18.7) |  |  |  |  | <0.001 |  |  |  | 0.720 |  | 0.177 |  |  |  | 0.789 |  |  | 0.775 |  | 0.083 |  |  |  | 0.001 |  | 0.902 |
| ≤75 | 135 | 135 | 14.0 | 9.8 |  | 3 | 1.7 | 1.2 |  | 2.22% |  | 8 | 1.4 | 0.7 |  | 13.1 | 14.0 |  | 5.93% |  | 100 | 55.3 | 108.4 |  | 74.07% |  |
| >75 | 427 | 427 | 18.2 | 10.2 |  | 21 | 1.5 | 0.8 |  | 4.92% |  | 47 | 1.5 | 0.9 |  | 14.7 | 14.4 |  | 11.01% |  | 314 | 101.8 | 176.2 |  | 73.54% |  |
| Compliance of inhalers (mean ± SD = 85.9 ± 21.8) |  |  |  |  | <0.001 |  |  |  | 0.720 |  | 0.094 |  |  |  | 0.567 |  |  | 0.974 |  | 0.024 |  |  |  | 0.006 |  | 0.835 |
| ≤75 | 153 | 153 | 14.9 | 10.0 |  | 3 | 1.7 | 1.2 |  | 1.96% |  | 8 | 1.6 | 0.9 |  | 14.6 | 14.4 |  | 5.23% |  | 112 | 68.1 | 133.2 |  | 73.20% |  |
| >75 | 405 | 405 | 18.2 | 10.2 |  | 21 | 1.5 | 0.8 |  | 5.19% |  | 47 | 1.4 | 0.9 |  | 14.4 | 14.4 |  | 11.60% |  | 300 | 99.4 | 173.4 |  | 74.07% |  |
| Clinical clusters |  |  |  |  | 0.010 |  |  |  | 0.963 |  | 0.958 |  |  |  | 0.702 |  |  | 0.513 |  | 0.078 |  |  |  | <0.001 |  | 0.080 |
| Cluster 1: smoking asthma | 37 | 37 | 14.0 | 8.5 |  | 1 | 1.0 | 0.0 |  | 2.70% |  | 3 | 1.0 | 0.0 |  | 17.7 | 24.5 |  | 8.11% |  | 22 | 33.7 | 33.7 |  | 59.46% |  |
| Cluster 2: severe obstructive asthma | 71 | 71 | 18.3 | 11.6 |  | 3 | 1.3 | 0.6 |  | 4.23% |  | 11 | 1.4 | 0.7 |  | 14.2 | 12.8 |  | 15.49% |  | 56 | 130.8 | 197.6 |  | 78.87% |  |
| Cluster 3: early-onset atopic asthma | 105 | 105 | 13.1 | 10.4 |  | 4 | 1.5 | 1.0 |  | 3.81% |  | 6 | 1.2 | 0.4 |  | 6.3 | 1.6 |  | 5.71% |  | 65 | 37.2 | 38.9 |  | 61.90% |  |
| Cluster 4: late-onset mild asthma | 106 | 106 | 14.3 | 9.4 |  | 5 | 1.4 | 0.9 |  | 4.72% |  | 6 | 1.2 | 0.4 |  | 16.8 | 15.3 |  | 5.66% |  | 72 | 31.7 | 53.3 |  | 67.92% |  |

Abbreviations: SD, standard deviation.

P-values were determined with a chi-square test, Student's *t*-test, or ANOVA.

Table S6 Asthma-related healthcare use according to adherence to the cohort

| **Variable** | **Total number  of  patients  (n = 736)** | **Outpatient visits** | | | | **Emergency department visits** | | | | | | **Hospitalizations** | | | | | | | | | **The use of systemic corticosteroids** | | | | | |
| --- | --- | --- | --- | --- | --- | --- | --- | --- | --- | --- | --- | --- | --- | --- | --- | --- | --- | --- | --- | --- | --- | --- | --- | --- | --- | --- |
|  |  | **Number  of  patients** | **Per capita frequency of outpatient visits** | | | **Number  of  patients** | **Per capita frequency of emergency department visits** | | | **Presence of  emergency visits** | | **Number  of  patients** | **Per capita frequency of hospitalizations** | | | **Per capita cumulative duration (days) of hospitalizations** | | | **Presence of hospitalizations** | | **Number  of  patients** | **Per capita cumulative duration (days) of systemic steroids use** | | | **Presence of systemic steroids use** | |
|  |  |  | **mean** | **SD** | **p** |  | **mean** | **SD** | **p** | **percent** | **p** |  | **mean** | **SD** | **p** | **mean** | **SD** | **p** | **percent** | **p** |  | **mean** | **SD** | **p** | **percent** | **p** |
| Cohort tracking status |  |  |  |  | <0.001 |  |  |  | 0.918 |  | 0.163 |  |  |  | 0.844 |  |  | 0.348 |  | 0.843 |  |  |  | 0.902 |  | <0.001 |
| Maintained in the cohort | 480 | 480 | 17.5 | 9.6 |  | 22 | 1.6 | 0.9 |  | 5% |  | 51 | 1.5 | 0.9 |  | 14.3 | 14.3 |  | 11% |  | 368 | 87.1 | 163.0 |  | 77% |  |
| Dropped out of cohort | 256 | 256 | 14.1 | 13.6 |  | 18 | 1.6 | 1.2 |  | 7% |  | 26 | 1.5 | 1.2 |  | 22.4 | 41.8 |  | 10% |  | 155 | 89.0 | 167.3 |  | 61% |  |
| Cohort tracking status in patients with FAO |  |  |  |  | 0.549 |  |  |  | 0.881 |  | 0.593 |  |  |  | 0.525 |  |  | 0.633 |  | 0.952 |  |  |  | 0.279 |  | 0.542 |
| Maintained in the cohort | 83 | 83 | 19.2 | 8.9 |  | 6 | 1.8 | 1.0 |  | 7% |  | 11 | 1.6 | 0.9 |  | 20.3 | 18.0 |  | 13% |  | 65 | 146.4 | 218.5 |  | 78% |  |
| Dropped out of cohort | 8 | 8 | 23.3 | 17.8 |  | 1 | 2.0 | . |  | 13% |  | 1 | 1.0 | . |  | 11.0 | . |  | 13% |  | 7 | 311.0 | 362.4 |  | 88% |  |
| Cohort tracking status in patients with RAO |  |  |  |  | <0.001 |  |  |  | 0.882 |  | 0.457 |  |  |  | 0.418 |  |  | 0.579 |  | 0.275 |  |  |  | 0.688 |  | <0.001 |
| Maintained in the cohort | 384 | 384 | 17.1 | 9.5 |  | 14 | 1.5 | 0.9 |  | 4% |  | 36 | 1.4 | 0.9 |  | 12.8 | 13.3 |  | 9% |  | 293 | 70.9 | 135.7 |  | 76% |  |
| Dropped out of cohort | 181 | 181 | 12.9 | 12.6 |  | 9 | 1.4 | 0.7 |  | 5% |  | 12 | 1.3 | 0.5 |  | 10.4 | 9.88 |  | 7% |  | 105 | 64.7 | 136.7 |  | 58% |  |

Abbreviations: SD, standard deviation.

P-values were determined with a chi-square test or Student's *t*-test
