# Supplementary material for: Risk Factors for Asthma-Related Healthcare Use: Longitudinal Analysis Using the NHI Claims Database in a Korean Asthma Cohort
Source: PLoS One. 2014 Nov 14;9(11):e112844. doi: 10.1371/journal.pone.0112844 (PMC4232512; doi:10.1371/journal.pone.0112844)
Supplement: Methods and Results S1 — Methods S1. SUPPLEMENT METHODS. Results S1. SUPPLEMENT RESULTS. (DOCX) [file pone.0112844.s004.docx]

**METHODS S1 (SUPPLEMENT METHODS)**

**Study population**

The present study used the COREA (COhort for Reality and Evolution of adult Asthma in Korea) data obtained by an asthma working group in Korea. This is a prospective, multicenter, 9-year (2005–2013), doctor-diagnosed asthma patient cohort. The patients were recruited by allergists or pulmonologists from 11 tertiary referral hospitals. The study subjects were 14 years or older and had been suffering for more than three months from asthma. Asthma was diagnosed by demonstration of RAO by the bronchodilator response (BDR) test or by airway hyperresponsiveness (AHR) following methacholine provocation.

**Measures**

*Baseline clinical characteristics*

The variables considered as baseline clinical characteristics were age, sex, body mass index (BMI), amount of smoking (pack-year), history of exacerbation, family history of allergic diseases, duration of asthma, presence of atopy, rhinosinusitis, and BDR test results, the forced expiratory volume in one second (FEV_1_)% predicted, blood eosinophil counts, and total serum IgE levels. For analyses, some variables were categorized as follows: the asthma duration was classified as more than 10 years or less than 10 years at the time of enrollment[[1](#_ENREF_1)]; age was split into 3 categories (<30, ≥30 and <60, ≥60)[[2](#_ENREF_2)]; pack-years was split into 2 categories (<10 and ≥10)[[3](#_ENREF_3)]; compliance of drug (inhaler) was seperated into 2 categories (≤75 and > 75)[[4](#_ENREF_4)]; blood and sputum eosinophilia were classified into 2 categories with 5% and 3% (respectively).[[5](#_ENREF_5)]

*Prescriptions*

Prescriptions for asthma medication were investigated at 3-month intervals during the index period, included ICSs, ICSs/LABAs, SABAs, oral leukotriene antagonists, and theophylline derivatives, and covered all forms of drug administration, such as oral medications, injections, and inhalations.

*Adherence to the cohort*

The adherence to the cohort was divided into 'maintained in the cohort' and 'dropped out of the cohort'. Patients were considered as 'dropped out of cohort' if they made no further outpatient visits to the initially enrolled hospitals after 12 months of cohort enrollment. The 'maintained in the cohort' group comprised the remaining study subjects.

**Statistical analysis**

Data were analyzed with the SAS statistical program (version 9.2; SAS Institute, Cary, NC, USA). First, frequencies of outpatient visits, hospitalization, and emergency department visits, duration of hospitalization, and systemic corticosteroid use, and the presence of hospitalization, emergency department visits, and systemic corticosteroid use were calculated to enable comparison of baseline clinical characteristics, clinical phenotypes, and adherence to the cohort with a chi-square test, Student's *t*-test, and analysis of variance (ANOVA). Second, multiple regression and multiple logistic regression were performed to predict the risk factors for asthma-related healthcare use, with adjustment for age, sex, and variables that had a p-value <0.1 in univariate models.

**RESULTS S1 (SUPPLEMENT RESULTS)**

**Overall asthma-related healthcare use**

During the index period, the most common number (in 173 patients) of outpatient visits was 11–15 (about 4–5 outpatient visits annually) (E Table 3). In addition, 10.5% (77/736) and 5.4% (40/736) required hospitalizations and emergency department visits, respectively, due to asthma exacerbation (Figure S1). In 81% (596/736) of patients, asthma-related outpatient visits were maintained in the 3^rd^ year. However, tracking in tertiary hospitals gradually decreased (82% in the 1^st^ year to 61% in the 3^rd^ year), whereas primary/secondary hospital visits increased (18% in the 1^st^ year to 39% in the 3^rd^ year). Hospitalizations and emergency visits were also increased over time in primary/secondary hospitals.

**Patterns of prescription of asthma medication**

ICS/LABA combinations in a single inhaler were used in 86.9% (640/736) of patients during the initial 3 months: 74.7% (550/736) were prescribed at tertiary referral hospitals, 0.1% (1/736) at primary/secondary hospitals, and 12.1% (89/736) at both primary/secondary and tertiary hospitals. Drug use continued to decrease, and eventually only 46% (339/736) used ICS/LABA in the last three months of the study: 30.8% (227/736) at tertiary referral hospitals, 8.8% (65/736) at primary/secondary hospitals, and 6.4% (47/736) at both primary/secondary and tertiary hospitals (Figure S2A). Prescription of ICS alone was maintained at 4.3–6.6% without major changes: 3.7–5.3% at tertiary referral hospitals, 0.0–0.5% at primary/secondary hospitals, and 0.1–0.9% at both primary/secondary and tertiary hospitals (Figure S2B). Prescription of oral leukotriene antagonists and theophylline derivatives, similar to that of ICS/LABA, gradually decreased: from 47.7% to 23.1% for oral leukotriene antagonists, and from 16.8% to 12.4% for theophylline derivatives (Figure S2C and 2D). SABA was prescribed to 21.3% of the patients in first three months, but use declined to form a plateau thereafter (7–9%) (Figure S2E). The percentage of patients prescribed systemic corticosteroids was 35.2% (259/736) during the first 3 months: 26.5% at tertiary hospitals, 0.3% at primary/secondary hospitals, and 8.4% at both primary/secondary and tertiary hospitals. Prescriptions slightly decreased thereafter, reaching 22.3% (164/736) in the last 3 months: 13.6% at tertiary hospitals, 3.9% at primary/secondary hospitals, and 4.8% at both primary/secondary and tertiary hospitals (Figure S2F).

**References**

1. Chetta A, Foresi A, Del Donno M, Bertorelli G, Pesci A, et al. (1997) Airways remodeling is a distinctive feature of asthma and is related to severity of disease. Chest 111: 852-857.

2. Boulet LP (1998) Perception of the role and potential side effects of inhaled corticosteroids among asthmatic patients. Chest 113: 587-592.

3. Polosa R, Russo C, Caponnetto P, Bertino G, Sarva M, et al. (2011) Greater severity of new onset asthma in allergic subjects who smoke: a 10-year longitudinal study. Respir Res 12: 16.

4. Jonasson G, Carlsen KH, Sødal A, Jonasson C, Mowinckel P (1999) Patient compliance in a clinical trial with inhaled budesonide in children with mild asthma. Eur Respir J 14: 150-154.

5. Gibson PG (2009) Inflammatory phenotypes in adult asthma: clinical applications. Clin Respir J 3: 198-206.
